# Good and Bad in the Hands of Politicians: Spontaneous Gestures during Positive and Negative Speech

**DOI:** 10.1371/journal.pone.0011805

**Published:** 2010-07-28

**Authors:** Daniel Casasanto, Kyle Jasmin

**Affiliations:** 1 Neurobiology of Language Group, Max Planck Institute for Psycholinguistics, Nijmegen, The Netherlands; 2 Donders Institute for Brain, Cognition, and Behaviour, Radboud University, Nijmegen, The Netherlands; 3 Department of Psychology, New School for Social Research, New York, New York, United States of America; University of Leuven, Belgium

## Abstract

**Background:**

According to the *body-specificity hypothesis*, people with different bodily characteristics should form correspondingly different mental representations, even in highly abstract conceptual domains. In a previous test of this proposal, right- and left-handers were found to associate positive ideas like *intelligence*, *attractiveness*, and *honesty* with their dominant side and negative ideas with their non-dominant side. The goal of the present study was to determine whether ‘body-specific’ associations of space and valence can be observed beyond the laboratory in spontaneous behavior, and whether these implicit associations have visible consequences.

**Methodology and Principal Findings:**

We analyzed speech and gesture (3012 spoken clauses, 1747 gestures) from the final debates of the 2004 and 2008 US presidential elections, which involved two right-handers (Kerry, Bush) and two left-handers (Obama, McCain). Blind, independent coding of speech and gesture allowed objective hypothesis testing. Right- and left-handed candidates showed contrasting associations between gesture and speech. In both of the left-handed candidates, left-hand gestures were associated more strongly with positive-valence clauses and right-hand gestures with negative-valence clauses; the opposite pattern was found in both right-handed candidates.

**Conclusions:**

Speakers associate positive messages more strongly with dominant hand gestures and negative messages with non-dominant hand gestures, revealing a hidden link between action and emotion. This pattern cannot be explained by conventions in language or culture, which associate ‘good’ with ‘right’ but not with ‘left’; rather, results support and extend the body-specificity hypothesis. Furthermore, results suggest that the hand speakers use to gesture may have unexpected (and probably unintended) communicative value, providing the listener with a subtle index of how the speaker feels about the content of the co-occurring speech.

## Introduction

Action and emotion are intimately linked in our everyday experiences. From infancy, people physically approach things they evaluate as *positive* and withdraw from things they evaluate as *negative*, a behavior that humans share with the simplest of organisms [1], [2]. Here we investigated whether the way people conceptualize and communicate ideas with positive and negative emotional valence is linked to the way they perform actions with their particular bodies.

Across languages and cultures, good things are conventionally associated with the *right*, and bad things with the *left*. This link is evident in English idioms with positive emotional valence like *the right answer* and *my right hand man*, and idioms with negative valence like *out in left field* and *two left feet*. The Latin words for right and left, *dexter* and *sinister*, form the roots of English words meaning skillful and evil, respectively. The words for right in French (*droite*) and in German (*Recht*) are closely related to the words meaning a ‘right’ or privilege accorded by the law, whereas the words for left in French (*gauche*) and German (*Links*) are related to words meaning distasteful or clumsy.

Links between left-right space and positive-negative valence are also found in nonlinguistic conventions. Roman orators were admonished never to gesture with their left hand, alone [3]. Likewise, in modern Ghanaian society, pointing and gesturing with the left hand is prohibited [4]. According to Islamic doctrine, the left hand should only be used for dirty jobs, whereas the right hand is used for eating. Likewise, the left foot is used for stepping into the bathroom, and the right foot for entering the mosque.

Why does good correspond to right and bad to left, throughout the world and throughout the ages? Left-right conventions in language and culture may arise as a consequence of ‘body-specific’ associations between action and valence. According to the *body-specificity hypothesis* [5]–[7], to the extent that the content of our minds depends on the structure of our bodies, people with different types of bodies should think differently, in predictable ways.

Bodies are lopsided. Most people have a dominant hand, usually the right hand [8], and therefore they interact with their environment more fluently on one side of body-centered space than the other. Greater perceptuomotor fluency has been shown to correlate with more positive evaluations: People like things that are easy to perceive and interact with [9], [10]. For example, expert typists prefer pairs of letters that can be typed easily over pairs that are more difficult to type (even when they’re not typing), suggesting that motor experience can influence affective judgments [11].

In a sense, we are all experts at using our dominant hands. Perhaps through asymmetrical motor experience, people come to implicitly associate good things with the side of their bodies they can use more fluently, and bad things with the side they use less fluently. On this proposal, linguistic and cultural conventions linking right with good may develop according to implicit handedness-based preferences of the right-handed majority.

If asymmetrical motor fluency causes people to develop associations between space and valence, then right- and left-handers should develop contrasting associations. For right-handers, right should be linked with good and left with bad, but the opposite association should be found in left-handers - at least implicitly - even though linguistic and cultural conventions suggest that everyone should associate right with good.

Laboratory experiments support this proposal. In one experiment, participants saw drawings of alien creatures sitting side by side on a page, and judged their ‘personal’ characteristics. On average, right-handers judged the aliens on the right to be smarter, happier, more honest, and more attractive, whereas left-handers judged the aliens on the left side more favorably. The same was true when right- and left-handers judged which of two products to buy or which of two job applicants to hire based on brief descriptions found on the left or right of a page. Right-handers tended to choose the person or product described on the right, but left-handers the person or product on the left [5].

**Figure 1:**
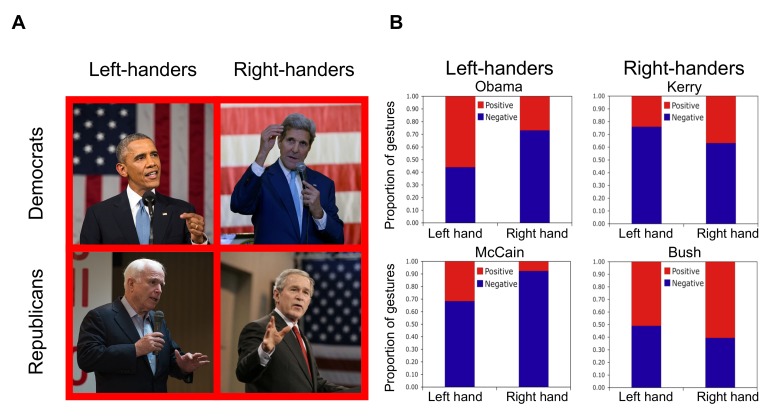
Examples of gestures and results of the speech-gesture analysis. **A. Examples of one-handed gestures produced by the 2004 and 2008 US presidential candidates.** Left panels: The left-handers, Obama (top) and McCain (bottom), gesturing with their left hands during speech with positive valence. Right panels: The right-handers, Kerry (top) and Bush (bottom), gesturing with their right hands during speech with positive valence. Due to copyright restrictions, the photos used here are for illustrative purposes only, and are not from the 2004 and 2008 US presidential debates that were analyzed in this study.** B. Associations between speech and gesture in each presidential candidate.** Proportions of right- and left-hand gestures during spoken clauses with positive (red) and negative (blue) emotional valence. In left-handers, left-hand gestures were more strongly associated with positive-valence speech than right-hand gestures, and right-hand gestures were more strongly associated with negative-valence clauses than left-hand gestures, but the opposite association between hand and valence was found in right-handers (Wald *χ*^2^=12.65, df=1, p=.0004). (Image sources: Official White House photography).

Here we investigated whether the body-specific association between people's dominant and non-dominant sides and ideas with positive and negative emotional valence can be observed beyond the laboratory, where people are not constrained to make binary choices. To test this association in spontaneous behavior, we analyzed a large and widely available corpus of speech and gesture: the final US presidential debates from 2004 and 2008. Serendipitously, both of the candidates from 2004 were right-handed (John Kerry, Democrat; George W. Bush, Republican), and both candidates from 2008 were left-handed (Barack Obama, Democrat; John McCain, Republican; fig. 1a). Do speakers tend to gesture more with their dominant hands when talking about good things, and their non-dominant hands when talking about bad things?

To find out, we parsed the complete transcripts from both debates into clauses. All spoken clauses were rated as expressing ideas with positive, negative, or indeterminate emotional valence, by raters blind to the gestures that accompanied them. Gesture strokes during clauses with positive and negative valence were then coded as having been performed with the left hand, right hand, or both hands. We tested for associations between the hand used to perform each gesture (dominant, non-dominant) and the emotional valence of the co-occurring spoken clause (positive, negative).

## Results

Across speakers, there was a strong association between the valence of the spoken clauses (positive, negative) and the hand used for spontaneous co-speech gestures (dominant, non-dominant), according to a binary logistic regression stratified by candidate (Wald *χ*^2^ = 12.65, df = 1, p = .0004; fig. 1b). Dominant hand gestures were more than twice as likely to occur during clauses with positive valence, and non-dominant hand gestures during clauses with negative valence (Odds Ratio (OR) for the regression of handedness on valence = 2.28, 95% C.I. = 1.46−3.57). In both of the left-handed candidates, left-hand gestures were more strongly associated with positive-valence clauses, and right-hand gestures with negative-valence clauses; in both right-handed candidates, right-hand gestures were more strongly associated with positive-valence clauses, and left-hand gestures with negative-valence clauses. The association between hand and valence was found in the predicted direction for every candidate, and the strength of the predicted association not differ significantly across candidates (Breslow-Day test for the homogeneity of odds ratios, *χ*^2^ = 2.97, df = 3, *ns*).

Do gestures follow political party lines? In linguistic metaphors, political affiliations are often spatialized along a left-right axis: Democrats are on the left and Republicans on the right of the political spectrum. Yet according to the candidates' gestures, the implicit mapping from the left and right hands to valence varies according to bodily characteristics, not politics. Candidates from both parties showed a similar pattern as was found in the full analysis, and overall, the association of hand and valence remained significant when the effect of political party was controlled by conditional logistic regression (Wald *χ*^2^ = 4.43, df = 1, p = .01; odds ratio estimate = 1.56, 95% C.I. = 1.03−2.35). The implicit association of dominant hand gestures with positive valence is something that Democrats and Republicans appear to agree on.

## Discussion

Spontaneous gestures during the final 2004 and 2008 US presidential debates revealed a previously unattested pattern: Dominant-hand gestures were more strongly associated with speech about good things, and non-dominant-hand gestures with speech about bad things. It was not simply the case that people gestured more with their dominant hands. Rather, right- and left-handers also used their hands in contrasting ways when expressing ideas with positive and negative emotional valence, suggesting that they automatically activated contrasting associations between action and emotion.

These data corroborate the results of laboratory tests showing that people implicitly associate good things with their dominant side and bad things with their non-dominant side [5]. Previous tests used binary forced-choice responses, however, which limits the extent to which the results generalize. Spontaneous gestures show that implicit links between ‘dominant’ and ‘good’ are not limited to the simplified world of the laboratory, but also extend to a world as complex as that of presidential politics.

There is a surprising implication of these findings. The hand that speakers use for spontaneous gestures provides an index of their feelings about the content of the co-occurring speech. If listeners can track which hand a speaker uses to gesture, they may be able to receive subtle clues to the speaker's attitude toward the things they are talking about — albeit the clues are statistical, not absolute, and the listener must know the speaker's handedness to interpret them.

For some speakers, the hand-valence association may be dramatic enough to observe with the ‘naked eye’, particularly in the non-dominant hand, which often makes fewer gestures overall. In this sample, negative-valence clauses accompanied by non-dominant hand gestures outnumbered positive-valence clauses by a ratio of more than 2 to 1 for Obama, more than 3 to 1 for Kerry, and the ratio was 12 to 1 for McCain, who made only 13 unimanual gestures with his non-dominant hand in total, almost exclusively during negative-valence clauses (Table 1). The suggestion that, in general, the non-dominant hand may serve as a more sensitive index of speakers' attitudes is speculative. Indeed, for Bush, the predicted body-specific hand-valence association was carried by gestures with his dominant hand. Non-dominant hand gestures may be more informative to listeners in everyday settings, however, because gestures with the dominant hand are likely to be too numerous to analyze intuitively.

**Table 1:** Number of right- and left-hand gestures during clauses with positive and negative emotional valence.

Candidate	Valence of Clause	Left hand gestures	Right hand gestures	Total
Obama (Left-hander, Democrat)	Negative	29	38	67
	Positive	37	15	52
	**Obama Total**	**66**	**53**	**119**
McCain (Left-hander, Republican)	Negative	168	12	180
	Positive	78	1	79
	**McCain Total**	**246**	**13**	**259**
Kerry (Right-hander, Democrat)	Negative	16	108	124
	Positive	5	64	69
	**Kerry Total**	**21**	**172**	**193**
Bush (Right-hander, Republican)	Negative	19	59	78
	Positive	20	94	114
	**Bush Total**	**39**	**153**	**192**
	**Grand Total**	**372**	**391**	**763**

Could the association of hand and valence be an artifact of the temporal/numerical order in which speakers mentioned good and bad things? This is unlikely, for several reasons. First, we find no evidence in the transcripts that speakers tended to mention good things before bad (or vice versa), systematically. Second, we only analyzed gestures during clauses expressing ideas of a single valence: mixed valence clauses like “you take the good with the bad” were excluded, so gesture patterns cannot be explained as spatializing ideas of contrasting valence within a clause. Third, in our previous laboratory experiments testing for interactions of space, valence, and handedness [5], the temporal/numerical order of good and bad responses were counterbalanced, and the predicted interactions were found to be independent of order. Finally, numerous experiments show that temporal and numerical primacy are associated with the left side of space, that this mapping is stable within a culture, and crucially that this mapping does not vary with handedness [12]: the left-to-right spatial mapping of primacy and succession cannot explain the difference we observe between right- and left-handers.

Could the pattern of gestures be an artifact of the candidates' positions relative to one another, to the moderator, or to the audience? All gestures pointing to anything in the room or indicating anyone (other than the speaker's self) were excluded from the analysis (see Materials and Methods). Still, in principle, the candidates' relative positions could have influenced their gestures [13]. For example, speakers could have made more positive gestures toward the moderator, or more negative gestures toward their opponent. Fortunately, within each debate the two candidates had the same handedness, and were therefore predicted to show *the same* pattern body-specific gestures (which they did). The candidates were positioned symmetrically on either side of a midline, side-by-side facing the audience, with the moderator in between them. For one candidate the moderator and the opponent were on the left, and for the other candidate they were on the right; therefore whatever effect the location of the moderator and the opponent may have had on a speaker's gestures, the effect should have been *mirror reversed* between the two candidates in each debate due to their symmetrical positions, working against the experimental hypothesis.

Finally, is important to consider whether speakers were aware of the association between hand and valence in their gestures, and whether conscious awareness of gesturing with one hand or the other could account for these results. This is possible in principle, but unlikely for two reasons. First, although people rarely speak aloud without knowing that they are speaking, they often gesture without realizing that they are gesturing [14]. Thus, much of the time, the candidates may not have been aware that they were gesturing *at all* — let alone that they were gesturing so as to produce the observed valence-handedness relationships. Second, it is plausible that the presidential candidates could have received coaching on how to gesture; perhaps they were even acquainted with historical treatises on gesture during oratory, which suggest favoring the right hand, and using the left hand only to express bad things [3], [15]. Such coaching could potentially contribute to the pattern found in right-handers, but not in left-handers; there is no reason to suspect that left-handers were coached to display the opposite pattern — against the classical practices of orators, and against everyday linguistic and cultural conventions.

Where does the implicit association between hand and valence come from? If this association were based on linguistic or cultural conventions, then all of the speakers should have shown a similar ‘good is right’ bias. In English-speaking cultures and many others, linguistic and non-linguistic conventions associate the right with ideas and actions that are good or allowable, and the left with those that are bad or prohibited. But there appear to be no conventions that link left with good and right with bad (‘left-wing’ and ‘right-wing’ politics notwithstanding, since it varies between individuals whether liberal or conservative political views are considered good). Furthermore, people must participate in the same social conventions regardless of their handedness. Left-handers are not allowed to greet people with left-handed handshakes, or to refer to the correct answer as *the left answer*.

The observed links between handedness, space, and valence could either result from innate differences between right- and left-handers or from asymmetries in bodily experience; associations between space and valence could be formed as people interact with their environment more fluently using their dominant hand (often on their dominant side) and less fluently using their non-dominant hand (often on their non-dominant side) [5]. The overall pattern of associations cannot be predicted or accounted for in terms of idioms in language or culture.

This is not to suggest that language, culture, and body are unrelated. On the contrary, the prevalence of the ‘good is right’ mapping across languages and cultures could be a result of right-handers' predominance in the population: Linguistic and cultural conventions reflect the implicit body-specific preferences of the majority. Both enculturation and bodily experience could potentially explain the ‘good is right’ mapping shown here in right-handers' gestures, but only bodily factors can account for the ‘good is left’ mapping found in left-handers' gestures. As such, these results support the body-specificity hypothesis [5]–[7]: people with different bodily characteristics mentally represent and communicate ideas differently, even in an abstract domain such as emotional valence which may appear far removed from physical action. Like investigations of linguistic relativity and cultural relativity, tests of *bodily relativity* [5] can increase our understanding of the diversity of the human conceptual repertoire.

## Materials and Methods

### Materials

Written transcripts for the final debates preceding the 2004 and 2008 US presidential elections were obtained from the Commission on Presidential Debates <www.debates.org>. Videos of the 2004 and 2008 debates were obtained from <www.archive.org> and <www.msnbc.msn.com>, respectively. The handedness of candidates was determined from the online resources including <en.wikipedia.org/wiki/Handedness_of_Presidents_of_the_United_States>, and confirmed by inspection of pictures and videos of the candidates writing or throwing, from various online sources.

### Procedure

#### Coding of spoken text

The goal of the text analysis was to determine the emotional valence of each spoken clause. Complete transcripts for both debates were parsed into clauses by a trained linguist, who served as Coder 1 for subsequent analyses. All analyses of the spoken text were conducted based on the written transcripts. The coders were blind to the gestures that accompanied them.

Two independent coders read each debate in full, classifying the valence for each clause as either positive, negative, neutral, or indeterminate (*i.e*., ambiguous or mixed valence). There were 3012 clauses, in total. Of these, 1325 (44%) were classified as valenced (either positive or negative) by both coders. Coders assigned the same valence to 1292 of these clauses (for examples see Table 2); thus inter-coder agreement for valence was 98%. Only those positive and negative clauses for which both coders agreed were submitted to the gesture analysis (686 with negative valence, 606 with positive valence).

**Table 2:** Examples of spoken clauses corresponding to dominant-hand gestures by each candidate.

Candidate	Hand	Valence of clause	Utterance
Obama	Left hand	Positive	“You can keep your health insurance.”
Kerry	Right hand	Positive	“You wanna buy into it, you can.”
McCain	Left hand	Positive	“And she has ignited our party and people all over America.”
Bush	Right hand	Positive	“And they will continue to get their checks.”

#### Coding of gestures

The goals of the gesture analysis were first to determine which hand was used for each gesture that accompanied spoken clauses with positive and negative valence, and then to test for associations of emotional valence with use of the dominant and non-dominant hand. Coder 1 edited the audio-video recordings of the debates, creating brief clips corresponding to each of the 1292 clauses that had been identified as positive or negative: one clause per clip. Clips lasted from the onset of the first word to the end of the last word of each clause. Coder 1 performed a non-blind analysis of the gestures in each clause, viewing the clips in chronological order and listening to the corresponding speech, to ensure that the clips contained the correct verbal material. During 179 of the clauses (14%), no gestures were observed. During the other 1113 of the clauses (86%), at least one gesture was observed. The video clips of these clauses were analyzed further.

Coder 1 determined the number of distinct gestures (*i.e*., gesture phrases) in each clip, according to segmentation criteria described by [16], and coded the hand(s) used for each gesture stroke (the most meaningful phase of each gesture): left, right, or both hands. Of the 1113 clips, 397 (36%) contained more than one gesture, yielding a total of 1747 gestures. Of these, 920 gestures (53%) were bimanual, and therefore could not be interpreted with respect to the experimental predictions. For the remaining 827 gestures (47%), the strokes were performed with either the left or the right hand, only. These gestures were analyzed further. The rate of uni-manual gestures per clause was similar across candidates (Obama: 0.41; Bush: 0.58; Kerry: 0.51; McCain: 0.88).

Of these 827 gestures, one was excluded (.001%) because the speaker's gesture space was substantially occluded due to the camera angle. An additional 43 gestures (5%) were excluded because they were highly stereotyped finger-counting gestures. Finally, 20 pointing or indicating gestures were excluded (2%) because they made deictic reference to one of the other people or objects in the room, so the speakers' choice of hand may have been influenced by their locations. The remaining 763 gestures (92%) comprised a mixture of iconic, metaphoric, deictic (abstract and self-referential), and most commonly beat-like gestures. Associations between the valence of the spoken clauses and use of the dominant hand were tested in these gestures, based on Coder 1's judgments (Table 1).

To test the reliability of these judgments, Coder 2 performed a blind (or rather *deaf*) analysis of the gestures identified by Coder 1, coding the hand(s) used for each stroke without listening to the accompanying speech. Of the 1747 gestures observed, 500 (29%) were randomly selected for reanalysis by Coder 2, half from 2004 and half from the 2008 debate. Selected video clips were numbered, and the non-consecutive clips were given to Coder 2. This coder did not know whether gestures were produced during clauses with positive or negative valence, and could not determine their content from context. Therefore, the ‘deaf’ coding could not be influenced by the coder's knowledge of the experimental predictions. Inter-coder agreement was 97%.
