# Correction: Good and Bad in the Hands of Politicians: Spontaneous Gestures during Positive and Negative Speech

**DOI:** 10.1371/journal.pone.0225175

**Published:** 2019-11-07

**Authors:** The PLOS ONE Staff

## Notice of republication

An incorrect version of Fig 1 was published in error. This article was republished on October 29, 2019 to correct for this error. At the time of republication, the Fig 1 caption and Table 2 caption were updated to reflect the corrected material. Please download this article again to view the correct version.
